# Barbara A. Mortimer, PhD

**Published:** 2006-09-15

**Table:** 

IN MEMORIAM
**Barbara Mortimer, PhD**
April 16, 1960 – August 30, 2006
Northrop Grumman Senior Editor and Editorial Staff Program Team Leader
Preventing Chronic Disease
The staff of *Preventing Chronic Disease* extends its deepest sympathies to the family and friends of Dr. Barbara Mortimer.
We are deeply enriched by her contributions to this journal and by the life she lived.

